# Pre-exposure prophylaxis to prevent the acquisition of HIV-1 infection (PROUD): effectiveness results from the pilot phase of a pragmatic open-label randomised trial

**DOI:** 10.1016/S0140-6736(15)00056-2

**Published:** 2016-01-02

**Authors:** Sheena McCormack, David T Dunn, Monica Desai, David I Dolling, Mitzy Gafos, Richard Gilson, Ann K Sullivan, Amanda Clarke, Iain Reeves, Gabriel Schembri, Nicola Mackie, Christine Bowman, Charles J Lacey, Vanessa Apea, Michael Brady, Julie Fox, Stephen Taylor, Simone Antonucci, Saye H Khoo, James Rooney, Anthony Nardone, Martin Fisher, Alan McOwan, Andrew N Phillips, Anne M Johnson, Brian Gazzard, Owen N Gill

**Affiliations:** a MRC Clinical Trials Unit at UCL, London, UK; b HIV & STI Department, Public Health England Centre for Infectious Disease Surveillance and Control, London, UK; c The Mortimer Market Centre, Central and North West London NHS Foundation Trust, London, UK; d St Stephen's Centre, Chelsea and Westminster Healthcare NHS Foundation Trust, London, UK; e Claude Nicol Centre, Royal Sussex County Hospital, Brighton & Sussex University Hospitals NHS Trust, Brighton, UK; f Homerton University Hospital NHS Foundation Trust, London, UK; g Manchester Centre for Sexual Health, Central Manchester University Hospitals NHS Foundation Trust, Manchester, UK; h St Mary's Hospital, Imperial College Healthcare NHS Foundation Trust, London, UK; i Sheffield Teaching Hospitals NHS Foundation Trust, Sheffield, UK; j York Teaching Hospital and Hull York Medical School, University of York, York, UK; k Ambrose King Centre and Barts Sexual Health Centre, Barts Health NHS Trust, London, UK; l King's College Hospital NHS Foundation Trust, London, UK; m Guy's and St Thomas' NHS Foundation Trust, London, UK; n Birmingham Heartlands Hospital, Heart of England NHS Foundation Trust, Birmingham, UK; o 56 Dean Street, Chelsea and Westminster Hospital NHS Foundation Trust, London, UK; p Gilead Sciences Foster City, CA, USA; q University of Liverpool, Liverpool, UK; r Research Department of Infection and Population Health, University College London, London, UK

## Abstract

**Background:**

Randomised placebo-controlled trials have shown that daily oral pre-exposure prophylaxis (PrEP) with tenofovir–emtricitabine reduces the risk of HIV infection. However, this benefit could be counteracted by risk compensation in users of PrEP. We did the PROUD study to assess this effect.

**Methods:**

PROUD is an open-label randomised trial done at 13 sexual health clinics in England. We enrolled HIV-negative gay and other men who have sex with men who had had anal intercourse without a condom in the previous 90 days. Participants were randomly assigned (1:1) to receive daily combined tenofovir disoproxil fumarate (245 mg) and emtricitabine (200 mg) either immediately or after a deferral period of 1 year. Randomisation was done via web-based access to a central computer-generated list with variable block sizes (stratified by clinical site). Follow-up was quarterly. The primary outcomes for the pilot phase were time to accrue 500 participants and retention; secondary outcomes included incident HIV infection during the deferral period, safety, adherence, and risk compensation. The trial is registered with ISRCTN (number ISRCTN94465371) and ClinicalTrials.gov (NCT02065986).

**Findings:**

We enrolled 544 participants (275 in the immediate group, 269 in the deferred group) between Nov 29, 2012, and April 30, 2014. Based on early evidence of effectiveness, the trial steering committee recommended on Oct 13, 2014, that all deferred participants be offered PrEP. Follow-up for HIV incidence was complete for 243 (94%) of 259 patient-years in the immediate group versus 222 (90%) of 245 patient-years in the deferred group. Three HIV infections occurred in the immediate group (1·2/100 person-years) versus 20 in the deferred group (9·0/100 person-years) despite 174 prescriptions of post-exposure prophylaxis in the deferred group (relative reduction 86%, 90% CI 64–96, p=0·0001; absolute difference 7·8/100 person-years, 90% CI 4·3–11·3). 13 men (90% CI 9–23) in a similar population would need access to 1 year of PrEP to avert one HIV infection. We recorded no serious adverse drug reactions; 28 adverse events, most commonly nausea, headache, and arthralgia, resulted in interruption of PrEp. We detected no difference in the occurrence of sexually transmitted infections, including rectal gonorrhoea and chlamydia, between groups, despite a suggestion of risk compensation among some PrEP recipients.

**Interpretation:**

In this high incidence population, daily tenofovir–emtricitabine conferred even higher protection against HIV than in placebo-controlled trials, refuting concerns that effectiveness would be less in a real-world setting. There was no evidence of an increase in other sexually transmitted infections. Our findings strongly support the addition of PrEP to the standard of prevention for men who have sex with men at risk of HIV infection.

**Funding:**

MRC Clinical Trials Unit at UCL, Public Health England, and Gilead Sciences.

## Introduction

HIV is a disease of major importance in the UK, with an estimated 107 800 individuals with HIV at the end of 2013.^1^ Prognosis is excellent, but treatment is lifelong with an inexorable increase in costs to the National Health Service.^2^ Gay, bisexual, and other men who have sex with men are the most at risk of acquiring HIV in the UK.^1^ There has been no decrease in the numbers of new diagnoses reported each year for the past decade (3250 in 2013), and estimates suggest that HIV incidence has increased in this population.^3^ These trends have occurred despite increased HIV testing and a move towards earlier initiation of antiretroviral therapy, which renders most patients non-infectious.^4^, ^5^ Although HIV testing and promotion of condom use will always be core strategies for reducing risk, a more radical approach is needed for people who do not have HIV and whose condom use is inconsistent. One such approach is pre-exposure prophylaxis (PrEP), the provision of antiretroviral drugs before HIV exposure to prevent infection.

The biological efficacy of daily oral tenofovir-based regimens used as PrEP to reduce HIV acquisition has been established through randomised placebo-controlled trials including men who have sex with men,^6^ heterosexual individuals,^7^, ^8^ and intravenous drug users.^9^ One purpose of using placebo in these studies was to avoid confounding bias due to risk compensation, which occurs if individuals perceive themselves to be protected by PrEP and so become more likely to engage in riskier sexual practices.^10^, ^11^ If this effect exists, it could undermine the biological protection conferred by PrEP and its value as a public health intervention.^10^, ^12^, ^13^, ^14^

**Research in context**

**Evidence before this study**

We reviewed all randomised controlled trials of pre-exposure prophylaxis (PrEP) listed in the HIV Prevention Research & Development Database, which has comprehensive information on biomedical clinical trials of HIV prevention that are planned, ongoing, or completed. We identified several completed and ongoing placebo-controlled trials designed to assess biological efficacy and demonstration projects designed to facilitate implementation, but no open-label randomised trials that assess real-life effectiveness.

**Added value of this study**

PROUD is the first-open-label randomised controlled trial of PrEP, and used a pragmatic schedule and procedures to represent how PrEP would be used in routine clinical practice. Our results refute concerns that the effectiveness of PrEP would be compromised when used in clinical practice, and the reduction in HIV incidence exceeded that reported from any placebo-controlled trial. The incidence of HIV infection among men not on PrEP was high (nine cases per 100 person-years), implying that the offer of PrEP is likely to attract individuals who are most likely to benefit from it.

**Implications of all the available evidence**

A public health programme of PrEP could have a major role in preventing a condition that requires lifelong treatment and curtailing the HIV epidemic. Structural and financial barriers that might impede its implementation should be urgently addressed.

We designed the PROUD study (appendix) to assess the effectiveness of PrEP. The effectiveness was the net effect of efficacy, adherence, and any change in sexual behaviour as a result of PrEP. Here, we report the pilot phase, in which we assessed recruitment and retention to test the feasibility of a large-scale trial. However, the unexpectedly large number of HIV infections enabled us to present findings on the effectiveness of PrEP, as well as safety, adherence, and risk compensation.

## Methods

### Study design and participants

We did this pragmatic, open-label, randomised controlled trial at 13 sexual health clinics in England. Eligible participants were male at birth, were aged 18 years or older, had previously attended the enrolling clinic, had been screened for HIV and other sexually transmitted infections, were HIV negative by a routinely used assay in the previous 4 weeks or on the day of enrolment, and had reported anal intercourse without a condom in the previous 90 days and likely in the opinion of the participant to have anal intercourse without a condom in the next 90 days. We excluded participants with acute viral illness possibly due to HIV seroconversion, any contraindication to tenofovir disoproxil fumarate or emtricitabine, and those being treated with or with treatment indicated for hepatitis B infection. The study was reviewed and approved by London Bridge Research Ethics Committee. The study protocol is available online. All patients provided written informed consent.

### Randomisation and masking

We randomly assigned participants (1:1) to receive PrEP either starting at the enrolment visit (immediate group) or after a deferral period of 1 year (deferred group). The computer-generated randomisation list with variable block sizes (of four, six, and eight; stratified by clinical site) was prepared by one of the trial statisticians (DID) and incorporated within the database held at the coordinating centre. Randomisation was web-based and done by approved members of the research team at each clinic. Regular sexual partners were encouraged to enrol together and both partners allocated to the same group to minimise the possibility of drug sharing. Neither patients nor investigators were masked to the treatment allocation.

### Procedures

We used procedures that we envisaged for a public health PrEP programme, including the lack of a screening visit, and the use of HIV and sexually transmitted infection results collected at other clinics and during non-study visits. All laboratory investigations were done locally with routine assays in compliance with the UK standards for the management of sexually transmitted infections.^15^ These guidelines recommend urethral, rectal, and pharyngeal nucleic acid amplification tests for *Chlamydia trachomatis* and *Neisseria gonorrhoeae*, with culture for *N gonorrhoeae* as indicated; serology for syphilis; serology or nucleic acid assays for hepatitis B and C as indicated. The protocol did not stipulate the collection and storage of a baseline sample for HIV although this was routine practice in some clinics.

At the enrolment visit, baseline demographic, clinical, and sexual behavioural data were recorded. Participants were screened for sexually transmitted infections if they reported a new partner since their previous screen, and assessed for hepatitis B immunisation status. A rapid antibody point-of-care HIV test was done if no HIV antigen–antibody test had been done in the previous 4 weeks, and all participants had an HIV antigen–antibody test after randomisation. Interventions to reduce risk were offered according to routine practice at the clinic.

The PrEP regimen was a single daily tablet containing 245 mg of tenofovir disoproxil fumarate and 200 mg of emtricitabine (Truvada; Gilead Sciences, Foster City, CA, USA). Participants allocated to the immediate group were initially prescribed 30 tablets together with information about dosing and potential side-effects, including that maximum protection against HIV would be achieved only after reaching steady state concentrations (roughly 2 weeks, estimated from five half-lives of the intracellular drug concentration).^16^ A blood sample was obtained to measure serum creatinine. An appointment was made within 1 month, primarily as a safety and tolerability check, and to prescribe 90 tablets. The same procedures were followed when participants in the deferred group started PrEP. Follow-up is scheduled to continue until the final enrolled participant has completed 2 years in the study.

All participants were asked to attend clinic every 3 months. These visits included an HIV test and a screen for bacterial sexually transmitted infections. Hepatitis C screening was indicated if the participant reported injecting or snorting drugs, fisting, or the use of sex toys. Sufficient PrEP was prescribed to extend 1 month beyond the next quarterly appointment. A subsequent protocol amendment allowed 6 months of PrEP to be prescribed in exceptional circumstances—eg, travel overseas. Serum creatinine was checked yearly, but additional tests were triggered at intervening visits if more than a trace of protein was detected by urine dipstick and could not be explained by infection.^17^ Potential side-effects of study drug and discontinuations for a medical event were asked about at each visit. In the event of HIV seroconversion, the earliest available HIV-positive sample was tested for genotypic drug resistance, in accordance with UK guidelines.^18^

Participants were asked to complete monthly questionnaires and daily diaries about sexual behaviour and adherence to PrEP, either online or on paper. A more detailed questionnaire, including information on the number and type of sexual partners in the previous 90 days, was administered at enrolment and at yearly visits. Plasma concentrations of tenofovir were measured in a sample of 52 participants who reported that they were taking PrEP and who attended one of five sites on a day the laboratory was able to process the samples. We attempted to identify additional HIV and sexually transmitted infection results in participants lost to follow-up by searching electronic clinic records in other PROUD clinics.

### Outcomes

The primary outcome was time to accrual of 500 participants and retention. The secondary outcomes were HIV infection, safety, adherence, and risk compensation (see protocol). HIV infection was defined as a reactive HIV antigen–antibody test result (confirmed by the detection of HIV RNA), in participants without HIV infection at enrolment. Although retrospective testing of enrolment samples for HIV RNA was possible at some sites these results were not considered.

We included data up to and including the first test after 48 weeks or the closure date of the deferred group on Oct 13, 2014, whichever was earlier (the deferral phase). We censored person-years of observation at the date of the first reactive HIV test for participants who became infected, or the date of the last test for those who did not. We calculated expected person-years of observation assuming that participants had attended all study visits, as per the study protocol.

### Statistical analysis

PROUD was designed with a sample size of 5000 participants, powered to detect a 50% reduction in HIV incidence from 2·5 to 1·25 infections per 100 person-years. For the pilot study, we used an arbitrary 10% sample size of 500. Because of the unlikeliness of showing the effectiveness of PrEP in a pilot study, data were initially monitored by a single independent expert not masked to allocation. As it emerged that HIV incidence was much higher than anticipated, an independent data monitoring committee was set up in June, 2014. The committee regarded the difference between groups in rate of HIV infection (rate difference) as the key measure for public health policy, and adopted a lower 2·5% confidence limit greater than two infections per 100 person-years as a threshold for notifying the steering committee, although this was not a formal stopping rule.

Analyses included all participants according to their randomised allocation (intention to treat) apart from the exclusion of individuals with a reactive HIV antigen–antibody result at enrolment in analyses of HIV incidence (modified intention to treat). We compared incidence rates between the two groups by both the rate difference and the rate ratio. We calculated exact 90% CIs rather than 95% CIs because we were primarily interested in the lower confidence limit—ie, the minimal estimate of the effectiveness.^19^ We derived the number-needed-to-treat to directly avert (prevent or delay) one HIV infection from the reciprocal of the rate difference.^20^ All analyses used data collected during the deferral phase of the trial, up to the date of extraction on June 10, 2015.

We planned to assess individual-level adherence and longitudinal sexual behaviour, but the low proportion of participants who completed the monthly questionnaire and diary prevented us from doing so. We therefore reported overall prescriptions of trial drug and cross-sectional analyses of sexual behaviour on the basis of baseline and 1 year questionnaires only. We compared the number of different anal sex partners at 1 year in each group using a stratified test for trend,^21^ according to the category at the enrolment visit. We used logistic regression to analyse the probability of detecting a sexually transmitted infection during follow-up, adjusting for the number of screens (as a linear term). We did the statistical analyses with Stata (version 13.1).

The trial is registered with ISRCTN (ISRCTN94465371) and ClinicalTrials.gov (NCT02065986).

### Role of the funding source

Employees of the funders had a role in the design of the study, data collection, analysis, and interpretation, and writing the report. The corresponding author had full access to all the data in the study and had final responsibility for the decision to submit for publication.

## Results

**Figure 1:**
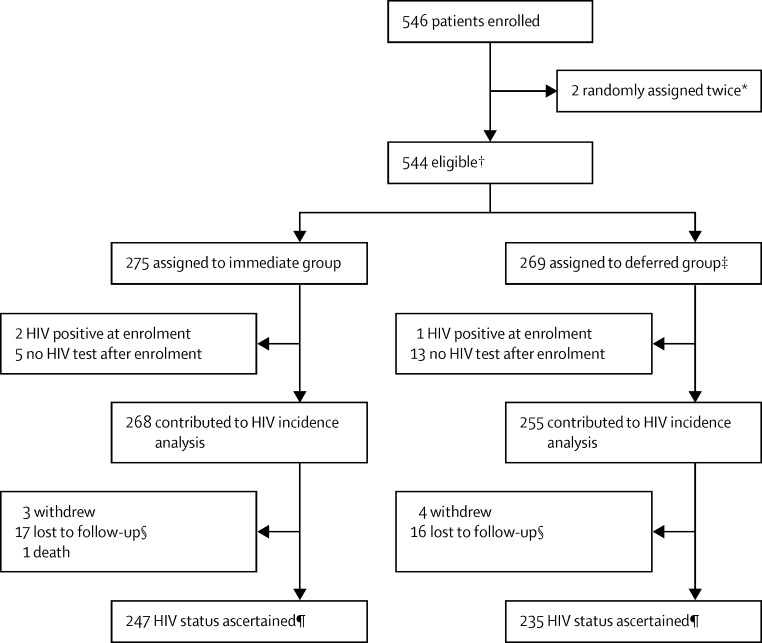
Trial profile *First to deferred and subsequently to immediate; considered in the deferred group for analyses but continued on pre-exposure prophylaxis. †19 pairs of partners were allocated to the same group (14 to immediate, five to deferred) including six pairs (all assigned to the immediate group) not enrolled concurrently. ‡One participant who was allocated to the deferred group was prescribed immediate pre-exposure prophylaxis in error; he was included in the deferred group for analyses but continued on pre-exposure prophylaxis. §Includes unable to contact, moved away, and non-attendance as no longer at risk. ¶HIV status ascertained if confirmed HIV-positive or HIV-negative test after 48 weeks or after Oct 13, 2014.

We randomly assigned 544 participants between Nov 29, 2012, and April 30, 2014: 275 to the immediate group and 269 to the deferred group. Two participants had enrolled twice to access PrEP and were analysed in the deferred group (figure 1).

The data monitoring committee considered the results of an interim analysis on Oct 6, 2014, and alerted the steering committee to a significantly increased risk of HIV infection in the deferred group. On Oct 13, 2014, the principal investigators at sites were requested by the steering committee to offer PrEP to all participants in the deferred group who had not yet had this opportunity (n=163).

Baseline characteristics were well balanced between the two groups (table 1). Median age was 35 years (29–43), 327 (61%) of 540 participants were university graduates, 217 (40%) of 540 were born outside of the UK, and 160 (30%) of 540 were living with a partner. In the previous 12 months, 331 (64%) of 517 had been diagnosed with a sexually transmitted infection (172 [33%] with rectal gonorrhoea or chlamydia), 184 (36%) of 510 had received at least one course of post-exposure prophylaxis, and the median number of HIV tests done was 3 (IQR 2–4). 231 (44%) of 525 participants had used one or more drugs associated with sexual disinhibition (γ-hydroxybutyrate, 4-methylmethcathinone, or methamphetamine) in the past 90 days.

14 (5%) of 275 participants in the immediate group were prescribed no further study drug after the initial prescription. Overall, sufficient study drug was prescribed for 88% of the total follow-up time. Tenofovir was detected in plasma of all 52 sampled participants (range 38–549 ng/mL) who reported that they were taking PrEP. 21 (8%) of 275 participants interrupted or missed doses because of 28 adverse event episodes. 13 of the episodes were considered related to study drug (table 2). 20 of 21 participants restarted study drug. The most common drug-related symptoms were nausea, headache, and arthralgia. Three of 21 participants interrupted study drug because of high creatinine concentration; two had comorbidities and were taking concomitant prescription drugs but a relationship to study drug could not be excluded, and one was thought to be due to recreational drugs. 29 serious adverse events (including one death) were reported in 27 participants, but none were attributed to study drug (appendix p 5).

Use of post-exposure prophylaxis (the recommended regimen at the time of the study was a 28-day course of tenofovir disoproxil fumarate–emtricitabine plus lopinavir) was common in the deferred group. 174 courses were prescribed to 85 participants during the deferral phase: 36 participants received one course, 27 received two courses, and 22 received three or more courses. Post-exposure prophylaxis was also prescribed to 12 participants (14 prescriptions) in the immediate group in this period.

**Figure 2:**
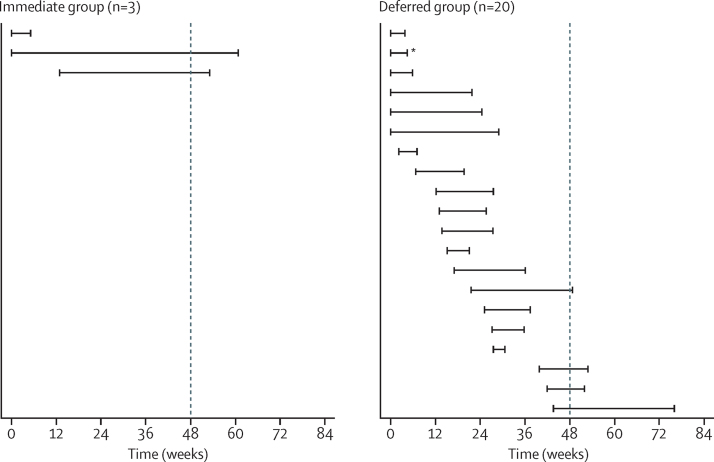
Incident HIV infections Left bound for each HIV case represents last non-reactive HIV test; right bound represents first reactive HIV test. The dotted line represents time when participants in the deferred group became eligible for pre-exposure prophylaxis under the original protocol. *Had a stored enrolment sample that tested positive for HIV RNA but was retained in the analysis.

Three participants (two in the immediate group, one in the deferred group) had a reactive HIV antigen–antibody test at baseline (all were non-reactive by an antibody point-of-care test; figure 1). A further 18 participants had no recorded HIV tests after the enrolment visit leaving 523 (96%) of 544 who contributed to the analysis of HIV incidence. We had 243 person-years of follow-up for the immediate group (94% of the expected 259 person-years) and 222 person-years for the deferred group (90% of the expected 245 person-years). 20 patients had new incident HIV infections in the deferred group (figure 2), of whom six had been prescribed a total of 12 courses of post-exposure prophylaxis during follow-up. In six patients, the last negative antigen–antibody test was at the enrolment visit. By contrast, only three incident HIV infections occurred in the immediate group. One participant had a reactive HIV test at the 4-week visit, and infection is thought to have pre-dated the start of PrEP, based on the history provided. The second participant was HIV reactive at 61 weeks and had been prescribed no study drug since the enrolment visit. The third participant presented with a seroconversion illness at 53 weeks; his last clinic attendance was at the 12-week visit when he was prescribed 90 tablets of study drug. These findings suggest that there were no breakthrough HIV infections in participants who were taking PrEP.

HIV incidence was significantly lower in the immediate group (1·2 cases per 100 person-years, 90% CI 0·4–2·9) than in the deferred group (9·0 per 100 person-years, 90% CI 6·1–12·8; p=0·0001). This difference corresponds to a proportionate reduction of 86% (90% CI 64–96) and a rate difference of 7·8 per 100 person-years (90% CI 4·3–11·3). 13 men (90% CI 9–23) in a similar population would need access to 1 year of PrEP to avert one HIV infection. HIV diagnoses in the deferred group were fairly evenly distributed over follow-up (appendix p 7).

All five participants in the immediate group who had HIV infection were tested for resistance. Two of the three participants with a reactive test at enrolment or the 4-week visit developed mutations at codon 184 in reverse transcriptase (Met184Ile/Met, Met184Ile/Val/Met), probably selected by exposure to emtricitabine. No resistance was detected in the two participants with later infections, which was not surprising given their apparent non-adherence to PrEP. No participant had mutations associated with tenofovir disoproxil fumarate treatment (Lys65Arg, Lys70Glu).

Questionnaires about sexual behaviour in the previous 90 days were completed and returned by 534 participants at baseline (271 in the immediate group *vs* 263 in the deferred group) and by 406 participants at 1 year (212 *vs* 194). Total number of different anal sex partners varied widely at the two timepoints, and we detected no significant difference between groups at 1 year (p=0·57; appendix p 8). However, a larger proportion of participants allocated to immediate PrEP than allocated to deferred PrEP reported receptive anal sex with ten or more partners without a condom (21% *vs* 12%; p=0·03, test for trend).

152 (57%) of 265 participants in the immediate group versus 124 (50%) of 247 in the deferred group were diagnosed with one or more bacterial sexually transmitted infection during follow-up, most commonly gonorrhoea and chlamydia (table 3). The randomised comparison was biased by the greater number of screens for sexually transmitted infections in the immediate group versus the deferred group (mean 4·2 *vs* 3·6), a consequence of more regular study clinic attendance to collect prescriptions in the immediate group. After adjustment for the number of screens, we found no significant difference between the groups, either for individual sexually transmitted infections or overall (table 3). Particularly, the proportion of participants diagnosed with rectal gonorrhoea or chlamydia, which is an indicator of receptive anal intercourse without a condom, was much the same in the two groups (table 3). Six incident hepatitis C infections occurred, three in each group. Injecting drug use was the possible route of transmission in three of these participants (two in the immediate group *vs* one in the deferred group), and a fourth participant (in the deferred group) acquired hepatitis C virus infection around the time or shortly after HIV infection.

## Discussion

Our findings refute concerns that the effectiveness of PrEP would be compromised in a real-world setting. Indeed, the reduction in HIV incidence we recorded exceeds that reported in any placebo-controlled trial.^22^ The proportion of sexually transmitted infections, including rectal gonorrhoea or chlamydia, did not differ significantly between groups despite a suggestion of risk compensation among a small proportion of PrEP recipients.

The study has strengths and weaknesses. First, the open-label rather than placebo-controlled design enabled us to capture the outcome that is most relevant for assessing PrEP within a public health prevention programme: the combination of the direct biological efficacy of the drug and the indirect effect of altered sexual behaviour among individuals who knew they were taking PrEP. Placebo-controlled trials may underestimate actual adherence because there is less incentive to take a tablet when the participant knows that it might be a placebo.^11^ Second, the lack of data on adherence to PrEP and sexual behaviour is a limitation. However, the measured drug concentrations validated the reports of participants who said they were taking study drug, by contrast with placebo-controlled trials.^6^, ^23^, ^24^ The absence of longitudinal data for sexual behaviour is frustrating, as we cannot assess precisely how participants matched adherence to risk, and insights into risk compensation are limited to a single timepoint at 1 year. However, we were able to use the information in the routine clinic records to capture the results of screens for HIV and sexually transmitted infection in the study database, and achieve a high level of follow-up for these endpoints. A larger study would have given more precise estimates of the effect of PrEP on sexually transmitted infections. Third, two men enrolled twice to access PrEP, and it is possible that others in the deferred group co-enrolled without detection or accessed PrEP from other sources, resulting in the effectiveness of PrEP being underestimated. Finally, because the trial stopped early the probability of type I error is increased.

An important issue in PrEP implementation programmes is eligibility.^22^ We included participants who reported at least one anal sex act without a condom in the preceding 90 days; consequently, the reported sexual risk behaviour at enrolment was diverse. Despite the broad eligibility criteria and extensive use of post-exposure prophylaxis, we recorded a high HIV incidence of nine cases per 100 person-years in the deferred group. This finding was the main determinant of the highly favourable estimate of 13 similar men who would need access to PrEP for 1 year to avert one HIV infection. Additional infections that would have occurred further down the transmission chain are not represented in this value. The incidence was roughly seven times higher than the national estimate of 1·34 cases per 100 person-years reported for men who have sex with men attending sexual health clinics in 2012, derived from avidity assay data.^25^ Although participants in PROUD were much more likely to have had rectal infections and to have used post-exposure prophylaxis than was the overall population of men who have sex with men attending sexual health clinics,^26^ the size of the difference in HIV incidence was nonetheless surprising. The difference suggests that the PROUD study population was highly selective, despite broad eligibility, and that the offer of PrEP generally attracts those men who are most likely to benefit from it. This finding is highly encouraging for PrEP implementation, although quantifying the likely demand in the UK remains challenging.

A potential disadvantage of PrEP is the generation of drug-resistant viruses and the resulting loss of treatment options.^27^ As was the case in the placebo-controlled trials,^22^ patients who had acute infection when PrEP was initiated had the highest risk of developing drug resistance. Acute infection can only be excluded if HIV testing follows a period of no potential exposure to HIV, which is not practical in people who have sex often and a delay in initiation of PrEP carries the greater risk of an HIV infection that could be avoided.

An economic assessment^28^ based on a mathematical model adapted to the UK epidemic in men who have sex with men suggests that providing targeted PrEP to this group from 2016 would be cost-effective at current prices, or without targeted implementation if tenofovir disoproxil fumarate–emtricitabine was halved in price. The investigators in the IPERGAY trial^29^ reported the same 86% reduction in HIV incidence using an on-demand regimen of tenofovir disoproxil fumarate–emtricitabine: two tablets taken 2–24 h before sex, one taken 24 h later, and one taken 48 h later. The median number of pills taken each month was 16, which would cost roughly half of a daily regimen. As well as fewer pills, other advantages of the on-demand regimen include the greater ease with which PrEP can be interrupted during periods of decreased or no risk.

In the UK, the standard of prevention is already high, with free walk-in services providing screening for HIV and sexually transmitted infections, treatment for sexually transmitted infections, condoms and encouragement to use them, post-exposure prophylaxis, and support for behaviour change. Nonetheless, there remains a substantial burden of new HIV diagnoses in men who have sex with men already attending sexual health clinics and thus accessing this standard of prevention. The impressive reduction in HIV incidence in people taking PrEP, without a measurable increase in other sexually transmitted infections, is reassuring for clinical, community, and public health stakeholders. National health services are under financial constraints, but they cannot afford to ignore the results of PROUD and IPERGAY, which strongly support the addition of PrEP to the current standard of prevention for men who have sex with men at risk of HIV infection.

## Figures and Tables

**Table 1:** Baseline characteristics

		**Immediate group (n=273)**	**Deferred group (n=267)**
Age (years)		35 (30–43)	35 (29–42)
Ethnicity			
	White	220 (81%)	219 (83%)
	Asian	14 (5%)	15 (6%)
	Black	11 (4%)	10 (4%)
	Other	28 (10%)	21 (8%)
University degree		161 (59%)	166 (62%)
Unemployed		24 (9%)	20 (8%)
Born outside the UK		110 (40%)	107 (40%)
Relationship status			
	Partner, living together	87 (32%)	73 (27%)
	Partner, living separately	40 (15%)	46 (17%)
	No partner	146 (53%)	147 (55%)
Circumcised		77 (28%)	79 (30%)
Chemsex* in past 90 days		115 (43%)	116 (45%)
Sexually transmitted infection diagnosed in past 12 months			
	Any	164 (63%)	167 (65%)
	Bacterial†	150 (58%)	155 (60%)
	Rectal gonorrhoea or chlamydia	89 (34%)	83 (32%)
Number of HIV tests in past 12 months		3 (2–4)	3 (2–4)
Used post-exposure prophylaxis in past 12 months		91 (35%)	93 (37%)

Data are median (IQR) or n (%). Two participants in each group did not return the questionnaire. Data were missing for ethnicity (none in the immediate group *vs* two in the deferred group), education (one *vs* none), employment status (none *vs* two), born outside UK (one *vs* none), relationship status (none *vs* one), circumcision status (two *vs* two), chemsex use (seven *vs* eight), history of sexually transmitted infection (13 *vs* ten), previous HIV tests (ten *vs* ten), and use of postexposure prophylaxis (15 *vs* 15).

* Use of either γ-hydroxybutyrate, 4-methylmethcathinone, or methamphetamine to facilitate or enhance sex.

† Gonorrhoea, chlamydia, or syphilis.

**Table 2:** Interruptions to treatment because of clinical or laboratory adverse events, by participant

	**Weeks since enrolment**	**Signs and symptoms**	Grade*	Relation to study drug*
A	44	Hospital-acquired pneumonia	Potentially life threatening	Unlikely
B	43	Chest pain musculoskeletal	Potentially life threatening	Unrelated
C	4	Headache	Severe	Probable
D	2	Fall	Severe	Unrelated
E	35	Anxiety or panic attack	Severe	Unrelated
F	43	Depression	Severe	Unrelated
G	52	Manic depression	Severe	Unrelated
H	0	Nausea, abdominal pain	Moderate	Probable
C	0	Headache	Moderate	Probable
I	5	Nausea	Moderate	Probable
J	24	Polyarthralgia	Moderate	Probable
K	49	Nausea	Moderate	Probable
L	0	Influenza-like illness	Moderate	Possible
M	4	High creatinine concentration	Moderate	Possible
H	1	Breathlessness, palpitations, chest pain	Moderate	Unlikely
N	1	Anxiety or depression	Moderate	Unlikely
O	1	Gastroenteritis	Moderate	Unlikely
H	2	Chest pain	Moderate	Unlikely
P	46	Loin pain	Moderate	Unlikely
B	47	Central chest pain	Moderate	Unlikely
Q	6	Headache	Moderate	Unrelated
O	6	Intermittent nausea	Mild	Definite
A	39	High creatinine concentration	Mild	Probable
R	12	Lipoatrophy	Mild	Possible
R	28	Fatigue, arthralgia	Mild	Possible
S	47	Arthralgia	Mild	Possible
T	5	High creatinine concentration	Mild	Unlikely
U	14	Abnormal liver function	Mild	Unlikely

Events in participants in the immediate group during the deferral phase of follow-up. All participants other than participant B restarted study drug.

* As assessed by participant's clinician.

**Table 3:** Bacterial sexually transmitted infections

	**Immediate**	**Deferred**	**Unadjusted odds ratio**	Adjusted odds ratio (90% CI)*	**p value**
Any	152/265 (57%)	124/247 (50%)	1·33	1·07 (0·78–1·46)	0·74
Gonorrhoea†	103/261 (39%)	89/242 (37%)	1·12	0·86 (0·62–1·20)	0·46
Chlamydia†	77/261 (30%)	54/242 (22%)	1·46	1·27 (0·89–1·80)	0·27
Syphilis	30/263 (11%)	22/247 (9%)	1·32	1·29 (0·79–2·10)	0·39
Rectal gonorrhoea or chlamydia	93/258 (36%)	77/238 (32%)	1·18	1·00 (0·72–1·38)	0·99

Infections diagnosed during deferral phase of follow-up. Analysis based on participants with at least one screen.

* Adjusted for the number of screens for specific infection.

† Detected in throat, urethra, or rectum.
