# Supplementary material for: Pre-exposure prophylaxis to prevent the acquisition of HIV-1 infection (PROUD): effectiveness results from the pilot phase of a pragmatic open-label randomised trial
Source: Lancet. 2016 Jan 2;387(10013):53–60. doi: 10.1016/S0140-6736(15)00056-2 (PMC4700047; doi:10.1016/S0140-6736(15)00056-2)
Supplement: Supplementary appendix [file mmc1.pdf]

THE LANCET

Supplementary appendix

This appendix formed part of the original submission and has been peer reviewed. We post it as supplied by the authors.

Supplement to: McCormack S, Dunn DT, Desai M, et al. Pre-exposure prophylaxis to prevent the acquisition of HIV-1 infection (PROUD): effectiveness results from the pilot phase of a pragmatic open-label randomised trial. *Lancet* 2015; published online Sept 10. http://dx.doi.org/10.1016/S0140-6736(15)00056-2.

SUPPLEMENTARY APPENDIX

This appendix contains supplementary material to:

Sheena McCormack, David T Dunn, Monica Desai, et al. Pre-exposure prophylaxis to prevent the acquisition of HIV-1 infection (PROUD): effectiveness results from the pilot phase of a pragmatic open-label randomised trial. Lancet. Published online September 10, 2015 http://dx.doi.org/10.1016/S0140-6736(15)00056-2 1

CONTENT

1. Study Contributors
2. Supplementary Table 1. Serious Adverse Events
3. Supplementary Table 2. Number of self-reported anal sex partners in past 90 days
4. Supplementary Figure 1. Supplementary Figure 1. Kaplan-Meier plot of time to first reactive HIV test
5. Supplementary Figure 2. Number of self-reported anal sex partners in past 90 days.

STUDY CONTRIBUTORS

PROUD clinic and research teams

Birmingham Heartlands Hospital: Sian Gately, Gerry Gilleran, Jill Lyons, Chris McCormack, Katy Moore, Cathy Stretton, Stephen Taylor, David White

Brighton and Sussex University Hospital: Alex Acheampong, Michael Bramley, Amanda Clarke, Martin Fisher, Wendy Hadley, Kerry Hobbs, Sarah Kirk, Nicky Perry, Celia Richardson, Mark Roche, Emma Simpkin, Simon Shaw, Elisa Souto, Julia Williams, Elaney Youssef

Chelsea and Westminster Hospital: Simone Antonucci, Tristan Barber, Serge Fedele, Chris Higgs, Kathryn McCormick, Sheena McCormack, Alan McOwan, Alexandra Meijer, Sam Pepper, Jane Rowlands, Gurmit Singh, Sonali Sonecha, Ann Sullivan, Lervina Thomas

Gilead Sciences: Andrew Cheng, Rich Clarke, Bill Guyer, Howard Jaffe, Hans Reiser, Jim Rooney,

Homerton University Hospital: Frederick Attakora, Richard Castles, Rebecca Clark, Anke De-Masi, Veronica Espa, Sifiso Mguni, Iain Reeves

King's College Hospital: Hannah Alexander, Jake Bayley, Michael Brady, Shema Doshi, Susanna Gilmour-White, Larissa Mulka, James Stephenson

Manchester Royal Infirmary: Brynn Chappell, Carolyn Davies, Dornubari Lebari, Matthew Phillips, Gabriel Schembri, Lisa Southon, Sarah Thorpe, Anna Vas, Chris Ward, Stephanie Yau

Mortimer Market Centre: Alejandro Arenas-Pinto, Asma Ashraf, Richard Gilson, Lewis Haddow, Ana Milinkovic, June Minton, Dianne Morris, Clare Oakland, Pierre Pellegrino, Sarah Pett, Carmel Young

MRC CTU at UCL: Sarah Banbury, Elizabeth Brodnicki, Christina Chung, Yolanda Collaco Moraes, David Dolling, David Dunn, Keith Fairbrother, Mitzy Gafos, Fleur Hudson, Sajad Khan, Shabana Khan, Sheena McCormack, Brendan Mauger, Mary Rauchenberger, Annabelle South, Yinka Sowunmi, Susan Spencer, Ellen White, Gemma Wood

Public Health England: Lucy Boxall, Monica Desai, Sarika Desai, Noel Gill, Kate Hyland, Anthony Nardone, Parnam Seyan

Sheffield Teaching Hospital: Anthony Bains, Gill Bell, Christine Bowman, Terry Cox, Matt Harrison, Charlie Hughes, Hannah Loftus, Naomi Sutton, Debbie Talbot, Vince Tucker

Social Science Team: Gill Bell, Mitzy Gafos, Rob Horne (Lead), Will Nutland, Caroline Rae, Michael Rayment, Sonali Wayal

Royal London Hospital: Vanessa Apea, Drew Clark, Paul Davis, James Hand, Claire Mayes, Margaret Portman, Liat Sarner, John Saunders, Angelina Twumasi, Wayne Smith, Salina Tsui, Avan Umaipalan, Ryan Whyte, Andy Williams

St Mary's Hospital: Wilbert Ayap, Adam Croucher, Olamide Dosekun, Kristin Kuldanek, Ken Legg, Nicola Mackie, Nadia Naous, Killian Quinn, Severine Rey, Judith Zhou

St Thomas's Hospital: Margaret-Anne Bevan, Julie Fox, Lisa Hurley, Helen Iveron, Isabelle Jendrulek, Tammy Murray, Alice Sharp, Chi Kai Tam, Al Teague, Juan Tiraboschi

York Teaching Hospital: Christine Brewer, Richard Evans, Jan Gravely, Charles Lacey, Fabiola Martin, Georgina Morris, Sarah Russell-Sharpe, John Wightman,

PROUD Governance (Independent members)

Trial Steering Committee: Michael Adler (Co-Chair), Gus Cairns (Co-Chair) Daniel Clutterbuck, Rob Cookson, Claire Foreman, Stephen Nicholson, Tariq Sadiq, Matthew Williams

Independent Data Monitor: Jack Cuzick

Independent Data Monitoring Committee: Simon Collins, Fiona Lampe, Anton Pozniak (Chair)

Community Engagement Group: Yusef Azad (NAT), Gus Cairns (NAM), Rob Cookson (LGF), Tom Doyle (Mesmac), Justin Harbottle (THT), Matthew Hodson (GMFA), Cary James (THT), Roger Pebody (NAM), Marion Wadibia (NAZ)

Supplementary Table 1. Serious Adverse Events

ID	Group	Weeks since enrolled	Signs/symptoms	Reason	Relationship to Truvada*
A	IMM	25	Severe stroke caused by vasculitis and high blood pressure which was complicated by pneumonia	Death	Unrelated
B	IMM	5	Variceal Bleed	Hospitalisation	Unlikely
C	IMM	19	Diarrhoea	Hospitalisation	Unlikely
B	IMM	39	Complications with ascites	Hospitalisation	Unlikely
B	IMM	44	Hospital acquired pneumonia	Hospitalisation	Unlikely
D	IMM	4	Fall	Hospitalisation	Unrelated
E	IMM	4	Panic attack/anxiety	Hospitalisation	Unrelated
F	IMM	12	Exacerbation of chronic pancreatitis	Hospitalisation	Unrelated
G	IMM	13	Diarrhoea/vomiting	Hospitalisation	Unrelated
H	IMM	13	Fracture (ankle)	Hospitalisation	Unrelated
I	IMM	16	Severe allergic reaction	Hospitalisation	Unrelated
J	IMM	25	Testicular torsion	Hospitalisation	Unrelated
K	IMM	36	Removal of metalwork	Hospitalisation	Unrelated
L	IMM	40	Anxiety/panic attack	Hospitalisation	Unrelated
M	IMM	44	Chest pain musculoskeletal	Hospitalisation	Unrelated
N	IMM	50	Attempted suicide and depression	Hospitalisation	Unrelated
O	IMM	50	Broken leg	Hospitalisation	Unrelated
P	IMM	53	HIV infection	Hospitalisation	Unrelated
Q	IMM	55	Acute diarrhoea, shigella	Hospitalisation	Unrelated
R	DEF	11	Acute hepatitis C	Hospitalisation	NA
S	DEF	13	Suicidal ideation	Hospitalisation	NA
T	DEF	15	Bacterial gastroenteritis	Hospitalisation	NA
U	IMM	22	Severe diarrhoea & vomiting	Hospitalisation	NA
V	DEF	37	Peri-anal abscess	Hospitalisation	NA
W	IMM	4	Fracture to head of radius	Persistent or significant disability or incapacity	Unlikely
X	DEF	34	Fractured right hand	Persistent or significant disability or incapacity	NA
Y	IMM	7	Hepatitis C	Any other important medical condition	Unlikely
Z	IMM	14	Chest pain	Any other important medical condition	Unlikely
AA	DEF	38	UTI/catheter replaced	Any other important medical condition	NA

Events on all participants during deferred phase of follow-up.
IDs are not the same as in Table 2
NA, not applicable
* As assessed by participant's clinician and reviewed by MD and/or SMc
Hospitalisation excludes elective procedures

Supplementary Table 2. Number of self-reported anal sex partners in past 90 days.

A. Total number of different anal sex partners irrespective of condom use (also displayed in Supplementary Figure 2).

**No. Partners**	**Enrolment**		**One year visit**	
	**IMM**	**DEF**	**IMM**	**DEF**
0	0 (0)	0 (0)	6 (3)	4 (2)
1	14 (5)	24 (9)	21 (10)	20 (10)
2-4	48 (18)	47 (18)	45 (21)	42 (22)
5-9	45 (17)	49 (19)	34 (16)	41 (21)
10-19	74 (27)	70 (27)	45 (21)	45 (23)
20+	90 (33)	73 (28)	61 (29)	42 (22)
**Total**	**271 (100)**	**263 (100)**	**212 (100)**	**194 (100)**

Values are number (%)

B. Number of different anal sex partners when participant was receptive and condom was not used (also displayed in Supplementary Figure 2)

**No. Partners**	**Enrolment**		**One year visit**	
	**IMM**	**DEF**	**IMM**	**DEF**
0	46 (18)	32 (12)	41 (20)	42 (22)
1	51 (20)	78 (30)	46 (22)	51 (26)
2-4	99 (38)	81 (32)	49 (23)	51 (26)
5-9	34 (13)	35 (14)	30 (14)	25 (13)
10-19	19 (7)	21 (8)	26 (12)	13 (7)
20+	9 (3)	10 (4)	18 (9)	11 (6)
**Total**	**258 (100)**	**257 (100)**	**210 (100)**	**193 (100)**

C. Number of different anal sex partners when participant was insertive and condom was not used

**No. Partners**	**Enrolment**		**One year visit**	
	**IMM**	**DEF**	**IMM**	**DEF**
0	46 (18)	37 (14)	45 (22)	38 (20)
1	58 (22)	69 (27)	46 (22)	40 (21)
2-4	78 (30)	65 (25)	37 (18)	52 (27)
5-9	37 (14)	50 (20)	41 (20)	32 (17)
10-19	29 (11)	21 (8)	25 (12)	18 (9)
20+	13 (5)	14 (5)	12 (6)	10 (5)
**Total**	**261 (100)**	**256 (100)**	**206 (100)**	**190 (100)**

Supplementary Figure 1. Supplementary Figure 1. Kaplan-Meier plot of time to first reactive HIV test

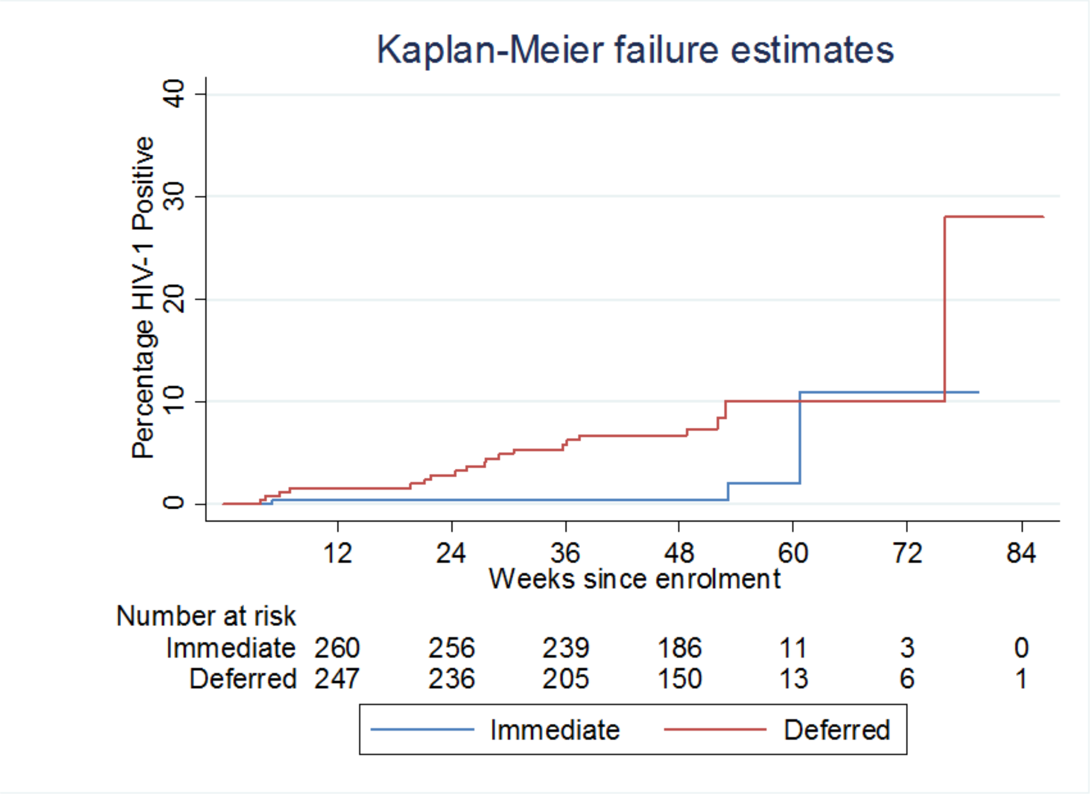

Supplementary Figure 2. Number of self-reported anal sex partners in past 90 days.

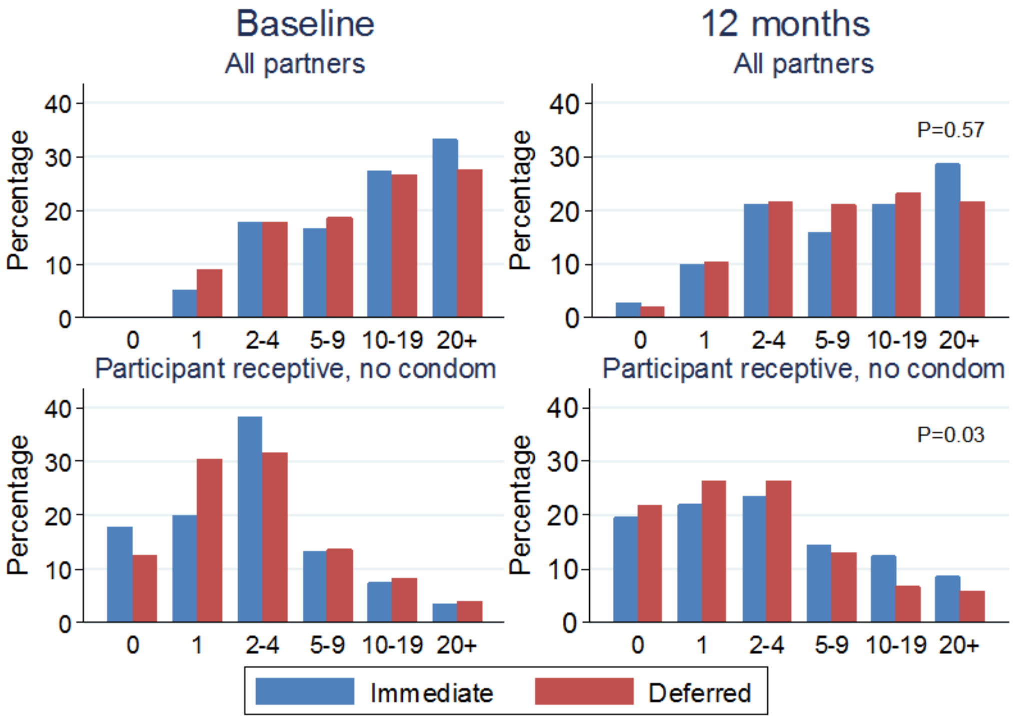

Top row, total number of different anal sex partners irrespective of condom use. Bottom row, number of different anal sex partners when participant was receptive and condom was not used. Left column, enrolment visit. Right column; visit at one year.
